# Dimensional Accuracy and Clinical Value of 3D Printed Models in Congenital Heart Disease: A Systematic Review and Meta-Analysis

**DOI:** 10.3390/jcm8091483

**Published:** 2019-09-18

**Authors:** Ivan Wen Wen Lau, Zhonghua Sun

**Affiliations:** Discipline of Medical Radiation Sciences, School of Molecular and Life Sciences, Curtin University, Perth 6845, Western Australia, Australia; ivan.w.lau@curtin.edu.au

**Keywords:** 3D printing, additive manufacturing, stereolithography, 3D model, congenital heart disease, congenital heart defect

## Abstract

The aim of this paper is to summarize and evaluate results from existing studies on accuracy and clinical value of three-dimensional printed heart models (3DPHM) for determining whether 3D printing can significantly improve on how the congenital heart disease (CHD) is managed in current clinical practice. Proquest, Google Scholar, Scopus, PubMed, and Medline were searched for relevant studies until April 2019. Two independent reviewers performed manual data extraction and assessed the risk of bias of the studies using the tools published on National Institutes of Health (NIH) website. The following data were extracted from the studies: author, year of publication, study design, imaging modality, segmentation software, utility of 3DPHM, CHD types, and dimensional accuracy. R software was used for the meta-analysis. Twenty-four articles met the inclusion criteria and were included in the systematic review. However, only 7 studies met the statistical requirements and were eligible for meta-analysis. Cochran’s Q test demonstrated significant variation among the studies for both of the meta-analyses of accuracy of 3DPHM and the utility of 3DPHM in medical education. Analysis of all included studies reported the mean deviation between the 3DPHM and the medical images is not significant, implying that 3DPHM are highly accurate. As for the utility of the 3DPHM, it is reported in all relevant studies that the 3DPHM improve the learning experience and satisfaction among the users, and play a critical role in facilitating surgical planning of complex CHD cases. 3DPHM have the potential to enhance communication in medical practice, however their clinical value remains debatable. More studies are required to yield a more meaningful meta-analysis.

## 1. Introduction

Congenital heart disease (CHD) is one of the commonest birth defects among newborns. Its morphology varies greatly across individuals and each case is unique in its own way, hence the planning of the corrective surgery for complex CHD is often very challenging [1,2]. However, a comprehensive understanding of the pathomorphology of complex CHD is difficult to achieve due to the shortcomings of current visualization techniques which are mainly based on cardiac computed tomography (CT), magnetic resonance imaging (MRI), and echocardiography imaging displayed and being interpreted on two-dimensional (2D) flat screens [3,4,5,6,7,8,9]. In order to prevent unexpected findings during surgery, it is imperative to have a precise comprehension of the spatial relationships between the intra-cardiac structures as well as the geometric relationships between the great vessels and surrounding anatomies [2,5,8,9,10].

Three-dimensional (3D) printing of anatomical models has shown to resolve the shortcomings of current visualization techniques [2,4,5,6,7,8,9,10,11,12,13,14,15,16,17,18,19,20,21,22,23,24,25,26,27,28,29,30]. It exploits the information from medical datasets and converts them into patient-specific 3D models that can be tangibly manipulated. As it manifests the cardiac structures in 3D views, observers can better interpret and gain a deeper understanding of the pathomorphology of complex CHD, thus helping surgeons to decide on the best surgical approach [6,8,12,22]. Despite the maturity of 3D printing technology in maxillofacial and orthopaedic fields, its application in the domain of CHD is still very limited [2,3,31,32,33]. Most of the published studies which investigated the application of 3D printed heart models (3DPHM; i.e., 3D printed anatomical models of the heart) are isolated case reports or case series that are largely anecdotal without strong statistical evidence, as shown in recent systematic reviews [3,34]. There are also no relevant meta-analyses to assess the dimensional accuracy as well as application of 3DPHM in the management of complex CHD. It remains unclear whether 3D printing can significantly improve on how the disease is managed in current clinical practice. Therefore, the aim of this systematic review and meta-analysis is to summarize and evaluate results from existing studies on accuracy and clinical value of 3DPHM.

Based on a previous systematic review, the current use of 3DPHM can be classified into five areas: Pre-operative planning, pre-surgical simulation, intraoperative orientation, medical education, and communication in clinical practice [34]. These will be considered the indicators to assess whether 3D printing can improve current clinical practice.

## 2. Methods

The systematic review and meta-analysis were strictly performed in accordance with the preferred reporting items for systematic reviews and meta-analysis (PRISMA) guidelines [35,36]. No ethics approval is required.

### 2.1. Search Strategy

A comprehensive and systematic literature search was performed in different databases including Proquest, Google Scholar, Scopus, PubMed, and Medline until April 2019. No constraint was applied to the publication date as the authors aim to conduct a comprehensive search on the topic of interest. Keywords like ‘congenital heart disease/defect’, ‘3D printing’, ‘rapid prototyping’, ‘additive manufacturing’, and ‘stereolithography’ were used along with Boolean operators to yield relevant search results. The exact search expressions were ‘congenital heart d*’ AND ‘3D print*’ OR ‘additive manufactur*’ OR ‘rapid prototyp*’ OR ‘stereolithograph*’. In order to increase the relevancy of the search results, the databases were set to only include studies that contain these keywords in the abstract, and to exclude review articles and case reports.

### 2.2. Study Selection and Eligibility Criteria

The title and abstract of the studies were screened for eligibility based on pre-designated inclusion and exclusion criteria. The inclusion criteria were as follows: (1) peer-reviewed articles, (2) published in English language, (3) used human data as subjects, (4) findings are based on patient-specific 3DPHM, and (5) studies contained at least one of the following indicators: Accuracy of the 3DPHM, evaluation of the usefulness of 3DPHM in planning surgeries or defining surgical approach, pre-surgical simulation, intraoperative orientation, medical education or training, and communication in clinical practice.

The exclusion criteria were as follows: (1) used non-human subjects, (2) studies about 3D bio-printing, (3) studies using virtual 3D heart models, and (4) case reports, reviews, commentary, or studies with only an abstract (or conference abstracts).

### 2.3. Data Extraction

The full-text of the studies that meets the eligibility criteria were sought. Data extraction was performed manually by two independent reviewers, who are also the authors of this review (IL and ZS). The following information was extracted from the chosen articles: Author, year of publication, study design, imaging modality, segmentation software, utility of 3DPHM, CHD types, and dimensional accuracy. Continuous variables were recorded in the form of arithmetic means and descriptive statistics if they were available in the study. The extracted data was tabulated in Microsoft Excel spreadsheet for analysis.

### 2.4. Quality Assessment

The quality of the included articles was appraised using the risk of bias tools published on the National Institutes of Health (NIH) website by the same two independent reviewers according to their study design [37]. Each item on the risk of bias tools was scored with ‘Yes’ and ‘No’. The number of ‘Yes’ was then tallied to decide if the article was of ‘good’, ‘fair’, or ‘poor’ quality. The author considered articles which scored ‘Yes’ in more than two-thirds of the questions as ‘good’ quality, more than one-third as ‘fair’ quality, and less than one-third as ‘poor’ quality.

### 2.5. Statistical Analysis

Statistical analyses were performed using R software, version 3.4.1 (http://www.r-project.org/), using the “metamean” and “metacont” functions in the R package *meta*, for single arm trials and two arms trials respectively. Single arm trials are studies which only consist of an experimental group, whereas two arms trials are studies consisting of both control and experimental groups. Cochran’s Q test was used to determine the homogeneity of the study means. Homogeneity of the study variances was tested with Bartlett’s test for the single arm trial. Null hypothesis (i.e., no statistical difference between the study data) is accepted only when p-values for both homogeneity tests are at least 0.05. Otherwise, the study data would not be pooled. The choice of ‘random effects model’ or ‘fixed effect model’ is determined by assessing whether there is any group structure present in the list of studies. If there is none, the fixed effect model is employed. 

## 3. Results

### 3.1. Literature Search

**Figure 1:**
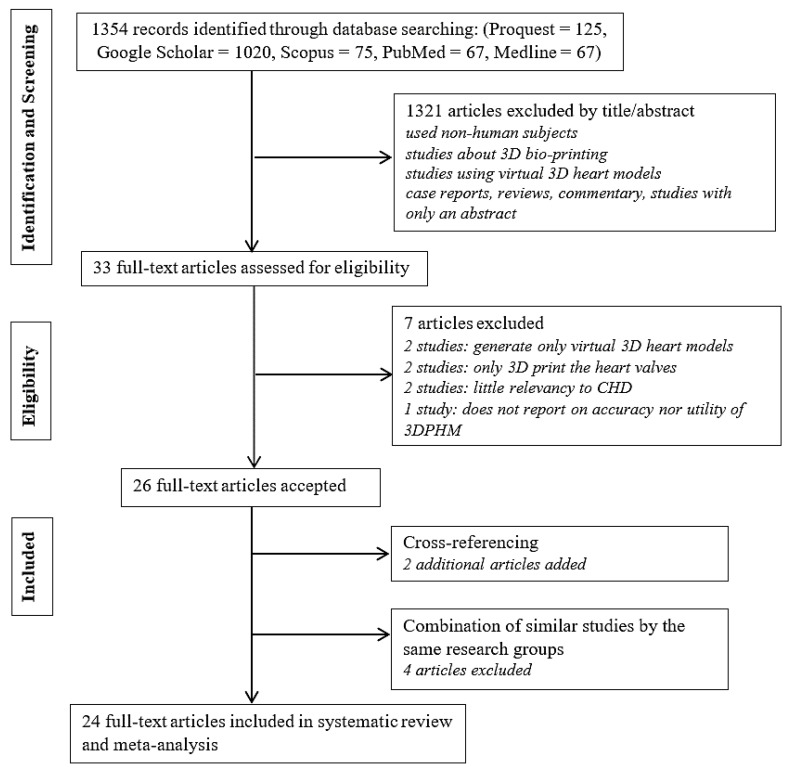
Flow chart of the study selection process. 3D, three-dimensional; 3DPHM, three-dimensional printed heart models; CHD, congenital heart disease.

The literature search retrieved a total of 1354 search results (125 from Proquest, 1020 from Google Scholar, 75 from Scopus, 67 from PubMed, and 67 from Medline). Following screening of the titles and abstracts resulted in exclusion of 1321 articles, as they were either non-relevant to the scope of review or did not meet the selection criteria. Full manuscripts were sought for the remaining 33 studies. Seven of them were further excluded for the following reasons: generation of virtual 3D models and non-relevant to 3D printing (*n* = 2), fabrication of only the heart valves (*n* = 2), not directly relevant to CHD (*n* = 2), and neither reported accuracy nor the utility of the 3DPHM (*n* = 1). Two additional articles were obtained from cross-referencing, resulting in a total of 28 selected articles. Among these publications, some were conducted by the same research groups. In order to remove bias in the results of this review, data from studies by the same research groups that are highly similar were combined and treated as one study. The similarities between these studies were investigated based on their study characteristics. This results in a total of 24 studies included in this systematic review and meta-analysis. Figure 1 outlines the study selection process.

### 3.2. Study Characteristics

**Figure 2:**
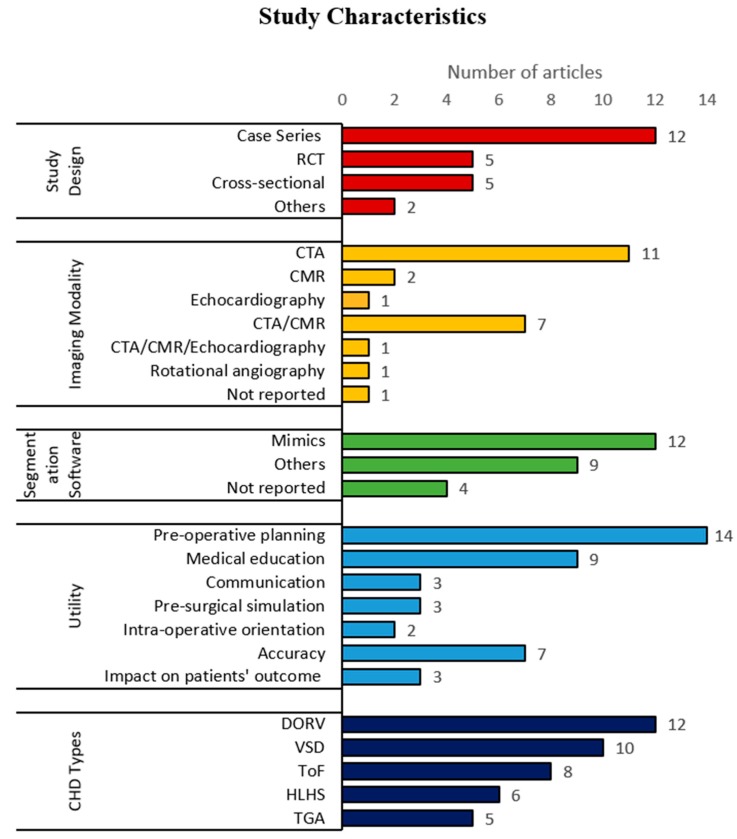
Horizontal histogram of the characteristics of the included studies. CHD, congenital heart disease; CMR, cardiac magnetic resonance; CTA, computed tomography angiography; DORV, double outlet right ventricle; HLHS, hypoplastic left heart syndrome; RCT, randomized controlled trial; TGA, transposition of great arteries; ToF, Tetralogy of Fallot; VSD, ventricular septal defect.

The characteristics of all included articles were summarized and are shown in Table 1 and Figure 2. Several publications that were conducted by the same research groups with similar characteristics (4 studies by Biglino et al. and 2 studies by Costello et al.) were combined and treated as one study, with only their most recent publication included in the systematic review. Table 1 highlights the 24 publications that are included in the review, whereas Figure 2 displays results with duplicates removed. 

As demonstrated in Figure 2, the study design employed for this research topic has a broad spectrum, however is mainly dominated by case series. CT angiography (CTA) is the predominant imaging modality used for generation of 3DPHM, followed by cardiac magnetic resonance (CMR), and echocardiography. There are a range of segmentation software tools used for cardiac segmentation, either open-source or commercial packages. Some studies utilized a mixture of different software for segmentation, in this case all of the software used in the study were counted and included in the frequency histogram in Figure 2. Materialise Mimics (Materialise HQ, Leuven, Belgium) is the mostly used software, with 12 out of 24 studies reporting its’ application for cardiac image segmentation. 

As mentioned before, 3DPHM have a range of applications. Some selected studies investigated more than one utility of the 3DPHM, in this case all of the utilities investigated are included in the count. Some studies reported important information on how the 3DPHM improve the patients’ outcomes (e.g. the length of cardiopulmonary bypass time, aortic cross-clamp time, mechanical ventilation time, duration of the surgery, patients’ readmission rate, length of hospitalization, and mortality rate), this type of information was classified under ‘impact on patients’ outcome’. It is found that pre-operative planning is reported as the primary utility of 3DPHM (Figure 2). The 3DPHM were generated for different types of CHD. Figure 2 lists the top five commonest types of CHD that were 3D printed. The most common type of CHD that has been 3D printed is the double outlet right ventricle (DORV). For the count for CHD types, the following rules apply:

1. In most of the cases, primary CHD is accompanied by secondary CHD. For example, DORV is usually accompanied by ventricular septal defect (VSD). In such cases, only the primary CHD is counted.
2. Each type of CHD is counted once per study, which means the number of cases per study does not contribute to the count.
3. CHD that have been repaired are also included in the count. For example, the study that produced 3DPHM of repaired TGA is counted.

### 3.3. Risk of Bias of the Included Studies 

Table 2 summarizes the quality of the 24 studies included in the review (good, fair, or poor). The scores of individual items in the assessment tool can be found in Supplementary Tables S1–S5. 

### 3.4. Meta-Analyses 

#### 3.4.1. Dimensional Accuracy of 3DPHM

**Figure 3:**
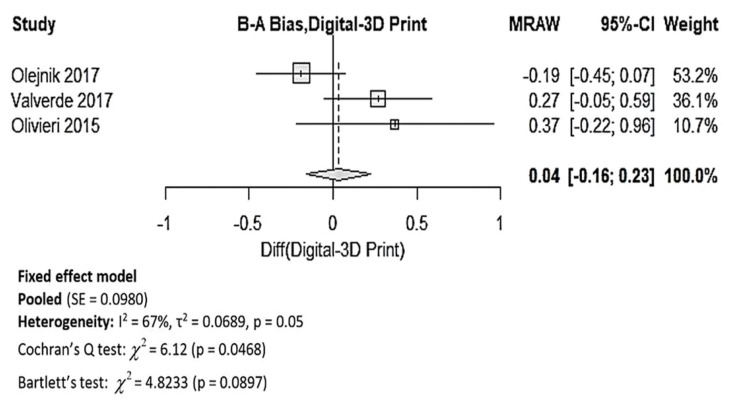
Forest plot for mean bias of the 3DPHM measurement and the digital images measurement. 3D, three-dimensional; B–A, Bland–Altman; MRAW, raw mean difference; CI, confidence interval; SE, standard error.

Upon data extraction, it was noticed that the data in the studies were presented very differently. Out of the 7 studies which reported the dimensional accuracy of 3DPHM [2,5,9,22,23,25,30], 3 studies reported only the correlation coefficient between the 3DPHM and the measurements based on the medical images, which is unsuitable for meta-analysis [2,25,30]. The other 4 studies provided means and standard deviations of the measurements, in which inverse variance weight of the individual study is able to be calculated [5,9,22,23]. Out of these 4 studies, one study compared the 3DPHM with in vivo surgical measurements, which is different from the other 3 studies that compared 3DPHM with digital images measurements [5]. As a result, only 3 studies were included in the quantitative synthesis of accuracy of 3DPHM [9,22,23], with mean bias and standard deviations (in millimeters) between the measurements of the 3DPHM and digital images as input data. Figure 3 demonstrates the forest plot generated for this meta-analysis. The pooled results demonstrated that the 3DPHM marginally underestimated the measurement, with a mean deviation of 0.04 mm, 95% CI (−0.16, 0.23) compared to the digital medical images. Cochran’s Q test demonstrates that there is significant variation among the mean bias (*p* = 0.0468). There is no evidence against the assumption of variance homogeneity (*p* = 0.0897). Despite so, the studies should not be pooled, as the first homogeneity test fails. 

#### 3.4.2. 3DPHM in Medical Education

**Figure 4:**
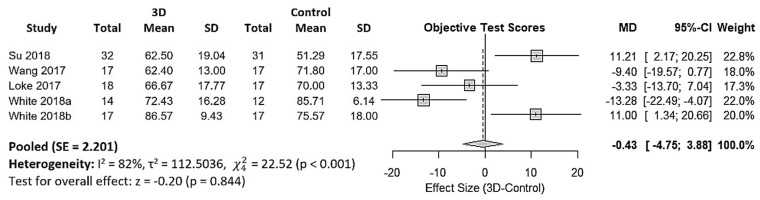
Forest plot for mean differences in test scores between the 3DPHM and the control groups. 3D, three-dimensional; CI, confidence interval; MD, mean difference; SD, standard deviation; SE, standard error.

Twelve studies reported utility of 3DPHM in medical education [2,9,12,13,14,15,16,19,24,27,28,29]. However, 7 of them used a Likert-type questionnaire to assess the gain in knowledge among the participants subjectively [2,9,12,13,14,15,24], and it is not known if the questionnaires used were tested for validity and reliability. Hence, this type of data is not suitable for quantitative synthesis. One study did not provide standard deviation of the data, hence was excluded from the meta-analysis [16]. This leads to 4 eligible studies for quantitative synthesis of 3DPHM in medical education [19,27,28,29]. These 4 studies are all two-arm trials which assessed the gain in knowledge among the participants by comparing test scores of the control group and 3DPHM group. The input data were converted to percentages as common unit for meta-analysis. Figure 4 is the forest plot generated for the meta-analysis. The pooled results demonstrated that the test scores in the 3DPHM group is lower than the control group, however this did not reach statistical significance (−0.43, 95% CI (−4.75, 3.88), *p* = 0.844). Cochran’s Q test demonstrates that there are significant variations among the mean test scores and the variances of the studies (*p* < 0.001). Hence, the studies should not be pooled.

## 4. Discussion

3D printing is an emerging new technology in the domain of cardiovascular surgeries. In spite of the increase in literature that suggested the benefits of this new technology, most of the studies remain as single-center experience with small sample size. There is also a lack of comprehensive systematic reviews and meta-analyses in the literature. A recent systematic review that was published in February 2019 provided an overview of the case studies available in the literature, mainly in the context of pre-operative planning [38]. However, there is neither inclusion nor exclusion criteria mentioned in the systematic review, nor are the steps conducted for the literature search. The comprehensiveness of this study remains questionable. This systematic review and meta-analysis fills in this research gap with a summary of six main utilities of 3DPHM in the context of CHD, which are pre-operative planning, pre-surgical simulation, medical education, intra-operative orientation, communication within clinical practice, and impact on patients’ outcome. To the best of our knowledge, this is also the first meta-analysis performed to analyze the accuracy and application of 3DPHM of CHD.

### 4.1. Dimensional Accuracy of 3DPHM 

The image acquisition technique and the image resolution are two main factors that can directly impact the dimensional accuracy of 3DPHM. CT is found to be the most preferred imaging modality for 3DPHM fabrication, as shown in Figure 2. 

The meta-analysis of the accuracy of 3DPHM demonstrates high heterogeneity amongst the three studies, hence the pooled mean deviation needs to be interpreted with care. The primary reason for this is likely due to the lack of eligible studies for analysis. It is also important to note that the analysis did not take into account the variability in segmentation methods, segmentation software and reconstruction protocol used, 3D printing methods, measuring methods, the imaging modality, image resolution, and the presence of artefacts in the source imaging data. All of the aforementioned factors can have notable influence on the dimensional accuracy of 3DPHM. Regardless of the heterogeneity, a mean deviation of 0.04 mm between the 3DPHM and medical images is considered negligible. This is because the 3D printing process works with tolerance ranges, and the model usually shrinks after curing of the polymer. Moreover, the image resolution for medical CT and MRI images will never be acquired in a resolution smaller than the range of deviation in daily clinical routine due to the high radiation dose from CT. Thus, mean deviation of 0.04 mm will not have any influence on the observation on the 3DPHM. Furthermore, all studies reported that the mean bias between the 3DPHM and the digital images are not significant, as indicated by the forest plot in Figure 3 (the confidence interval horizontal line for all three studies crosses over the vertical line). Additionally, all four studies that reported the correlation coefficient between the measurements of 3DPHM and the digital images indicated strong correlation (*r* > 0.98) [2,23,25,30]. All the findings from the studies are directed towards a conclusion that the 3DPHM are highly accurate.

### 4.2. 3DPHM in Medical Education

3DPHM were reported as a novel teaching approach when compared to the traditional way with 2D diagrams and sketches [19,29]. The forest plot may give the impression that 3D printing does not contribute to favorable outcomes in medical teaching. However, it is worth noting that White et al. studied the impact of 3DPHM in medical teaching for both simple and complex CHD, and they yielded very different results [29]. This is demonstrated in Figure 4 as ‘White 2018a’ and ‘White 2018b’. In the control group that attended VSD seminar (simple CHD), which only have access to images of the virtual models during the lecture, scored higher than the 3DPHM group in the post-lecture test. Interestingly, in the study group that attended Tetralogy of Fallot (ToF) seminar (complex CHD), the 3DPHM group scored higher than the control group. In both of the VSD and ToF cohorts, there are no significant differences in their baseline knowledge on CHD [29]. This finding suggests that 3DPHM play a role in teaching and learning of complex CHD that cannot be overlooked, although it might not be as useful in medical teaching for simple CHD such as VSD.

**Figure 5:**
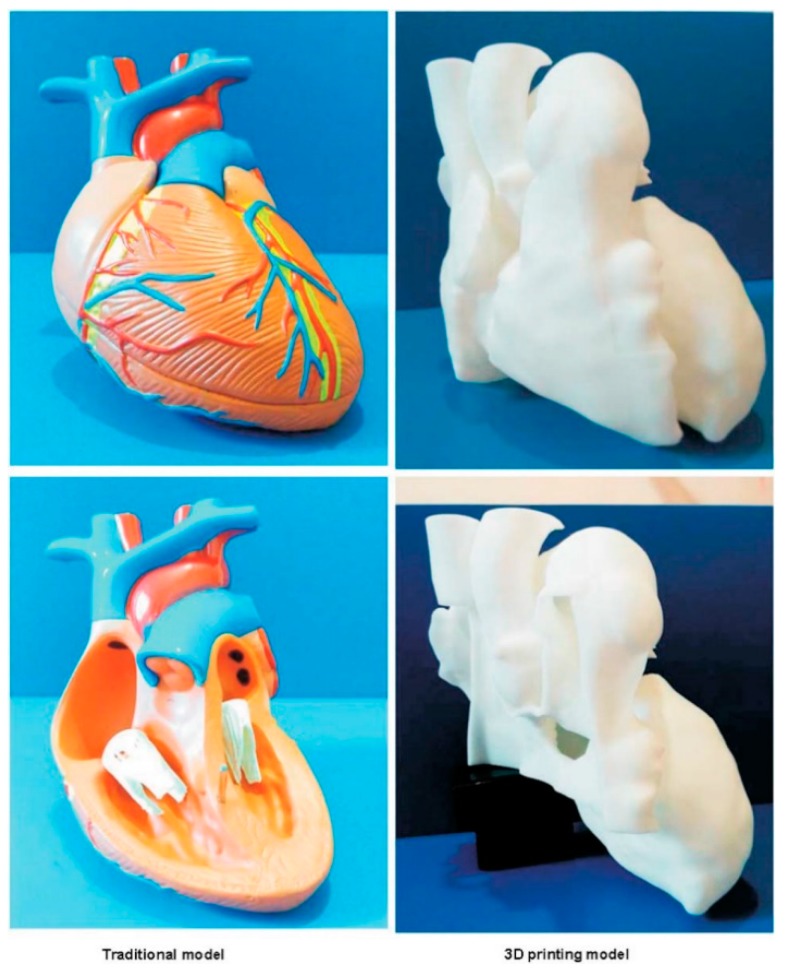
Traditional cardiac model (left) and 3D printed model (right) which are used in the study by Wang et al. for two different test groups to compare their role in facilitating medical education. Reprinted with permission under the open access from Wang et al. [28].

In another study by Wang et al., the author compared the impact of utilizing 3DPHM versus the traditionally made cardiac model in medical teaching (Figure 5) [28]. Their finding indicated that there is no significant improvement in the test scores among the 3DPHM group. However, the study did not have adequate bias control measures. First, the 3DPHM generated in the study features a simple VSD, whereas the traditional cardiac model demonstrates normal cardiac anatomy. Such difference in the teaching tools already made the two groups incomparable. Second, the medical questions asked in the test do not pertain to the pathology demonstrated on the 3DPHM. The lecture and the test questions in the study were designed for valvular heart diseases, thus in such case, a model with VSD was not the best teaching tool for the pathology of interest. 

Nevertheless, the findings from the selected studies indicate that 3DPHM can improve the learning experience and satisfaction. This is evidenced in all 12 studies that reported utility of 3DPHM in medical education, with the increased subjective evaluation scores and satisfactory level among the participants in the 3DPHM group [2,9,12,13,14,15,16,19,24,27,28,29]. In a study that involved 14 clinicians, medical education was ranked as the most relevant utility of 3DPHM. The 3DPHM were also described as more informative than the 2D diagrams [12]. However, due to the subjective nature of the evaluation, the results must be interpreted with care as they are more vulnerable to bias.

### 4.3. 3DPHM in Pre-Operative Planning

There are 15 studies that assessed the utility of 3DPHM in pre-operative planning. Out of these articles, 12 are observational and descriptive studies in which the surgeons utilized the 3DPHM to plan the surgical procedures before the surgery [6,7,8,9,10,12,16,17,18,20,21,22,26,30]; whereas the other 3 studies are quantitative studies that reported surgeons’ opinion on 3DPHM in pre-operative planning [2,12,16]. In most of these studies, 3DPHM were only generated when the surgeons could not fully understand the patients’ heart anatomy and needed further clarification [6,7,9,10,17,22]. It was reported that the tangible models have helped the surgeons to appreciate the complexity of the anomalous cardiac anatomy better, and even to help them visualize abnormalities that could not be clearly identified on the conventional cardiac images [9,21]. This has assisted the surgeons in defining the best surgical approach for the patients. It is important to note however, that the results from these studies only apply to complex CHD, as the fabrication of 3DPHM were mostly based on surgeons’ requests to obtain additional information. This is also evidenced in the finding of this review, with DORV as the commonest type of CHD that have been 3D printed. DORV is considered as complex CHD, as it is most often accompanied by a broad spectrum of anomalies. Hence, the perceived clinical value of 3DPHM demonstrated in these studies may not be the same when it comes to simple CHD [9].

In a multi-center study involving 10 international hospitals, it was reported that the 3DPHM acted as the deciding factor to alter the surgical decision in 19 out of 40 cases [9]. All 40 cases were assessed twice by the surgeons for operative planning, in which the first evaluation was based on conventional imaging data with virtual 3D reconstruction, and the second evaluation with the 3DPHM. The surgical plans derived from both of the evaluations were then compared. Three patients who were originally considered ineligible to undergo surgery based on conventional operative planning, were identified as surgical candidates based on 3DPHM planning and underwent successful surgical correction. The 3DPHM provide additional spatial information of the cardiac anatomy which is difficult to obtain from the 2D screen display, allowing the surgeons to appreciate potential surgical complications and modify the approach if necessary [9]. 

In another study conducted by Ryan et al., 33 cases with 3DPHM generated were studied retrospectively with regards to their 30-days admission and mortality rate, as well as the duration of surgery [26]. These results were compared with another 113 cases with similar types of lesions which received routine operative planning. The findings demonstrated an overall reduction in mean operative time for the 3DPHM group. These results echo with the findings from another study by Zhao et al., who compared 8 cases in the 3DPHM group with 17 cases in the control group [30]. The 3DPHM group had a much shorter operative time, cardiopulmonary bypass time, aortic cross-clamping time, and mechanical ventilation time than the control group. The findings in both of these studies implicitly indicate that the 3DPHM play a critical role in enhancing pre-operative planning, with a possible added value to reduce the costs for surgery following the reduction in duration of surgery. However, it is important to note that both of these findings did not achieve statistical significance. Insufficient statistical powering is more likely to be due to small sample size, rather than non-favorable outcomes [26].

### 4.4. 3DPHM in Communication within Medical Practice

All of the studies that reported the utility of 3DPHM in communication are questionnaire-based studies. The targeted population is variable, with 3 studies targeted at clinicians, radiologists, surgeons, and cardiologists [2,9,12], whereas the other 2 targeted at patients and parents [11,13]. From the health professionals’ perspective, both the studies by Valverde et al. and Lau et al. yielded very similar results where most of the participants agreed that the 3DPHM are useful in enhancing communication with other colleagues as well as patients and parents [2,9]. In a randomized controlled trial (RCT) in which 3DPHM were used during the consultation with parents, the cardiologists remarked that the use of 3DPHM resulted in a better interaction with the parents. This is evidenced by a 5-minutes-longer consultation duration on average in the 3DPHM group when compared to the control group (21 ± 10 vs. 16 ± 7 min, *p* = 0.02), which implicitly indicated that the 3DPHM stimulated curiosity, resulting in a more detailed discussion among the doctors and parents [11]. Interestingly, in another study by the same research group which involved 14 clinicians, communication was ranked as the least relevant utility compared to medical teaching, pre-operative planning, and research. However, this should not be perceived as 3DPHM is unbeneficial in improving communication, as 5 clinicians still ranked communication as the most relevant utility of 3DPHM [12].

From the non-professionals’ (patients and parents) point of view, most of them are very satisfied with the 3DPHM used in their consultation [11,13]. Despite their satisfaction, there was no significant increase in short-term knowledge acquisition among the experimental group of 45 parents [11]. Surprisingly, there was even a decrease in the cardiologist-assessed parental knowledge compared to the control group of 53 parents (7.0 ± 1.9 vs. 8.0 ± 1.7) [11]. Another study carried out by the same group of researchers but with adolescent patients as the participants yielded result that is vastly different. There is a significant objective increase in knowledge acquisition in the 3DPHM group (*p* < 0.001) as well as a subjective increase in their confidence in explaining their heart condition to others [13]. Nevertheless, a minor group of patients in the 3DPHM group (30%) reported increase in anxiety level after their consultation [11]. 

Instead of replacing the traditional approach that is to communicate based on medical images, 3DPHM seem to be acting as a complementary tool in the patient-doctor communication. In the study by Lau et al., the health professionals were asked if they prefer the 3DPHM or digital images to communicate with the patients or their colleagues. Sixty-seven percent of the participants indicated that they prefer to use both as a medium for communication [2]. This aligns with the finding in another study by Biglino et al., in which the teenage patients prefer to have digital simulations shown on the monitor in addition to the 3DPHM [12].

The clinical value of 3DPHM in enhancing communication within clinical practice remains arguable. Further research based on larger sample size from different stakeholders is warranted to holistically study the impact of 3DPHM on communication improvement.

### 4.5. Limitations 

The findings of this systematic review and meta-analysis are subjected to several limitations. First, even though there are 28 articles in total that met the inclusion criteria, only 24 were included in the final review, due to the similarities between studies by the same research groups. Out of the 24 studies, only 7 studies met the statistical requirements and are eligible for meta-analysis. One of the main reasons is because most of the studies are case series and did not provide quantitative data which is required for meta-analysis. However, this is due to case series being the dominant study design in the current literature, rather than a problem in the study selection process. Another reason is the lack of common outcomes assessment methods, which impede the authors from grouping the results for data synthesis. One solid example would be the measurement of the operative time to investigate if the use of 3DPHM in pre-operative planning can result in a reduction of surgery duration. Both the studies by Ryan et al. and Zhao et al. reported the operative time, however Zhao et al. did not define whether the operative time measured includes the time used to transport patients in and out of the operating theatre [26,30]. Second, the meta-analyses in this study demonstrated heterogeneity among the selected studies. This indicates that the study results should not be pooled. The pooled mean differences shown in the forest plot should be interpreted very carefully. This high heterogeneity is most likely due to the lack of studies eligible for meta-analysis. 

## 5. Conclusions and Implications of Future Work

Despite the limitations, this systematic review has analyzed the current literature with results that can be used to guide further research in this field. The results demonstrate that 3DPHM are dimensionally accurate. However, data from more studies are required to measure the mean deviation of the 3DPHM from the medical images measurement.

Even though 3DPHM might not increase the users’ short-term knowledge on CHD, it was reported to improve the learning experience and satisfaction level among the users. Future studies should aim to investigate the long-term impact on the knowledge acquisition among different stakeholders, such as students, patients and parents, and junior doctors.

Meta-analysis of the utility of 3DPHM in pre-operative planning was not possible in this study, due to the nature of the data being difficult to quantify. Nevertheless, the finding of the review suggests that 3DPHM play an important role in facilitating pre-operative planning of complex CHD cases, especially in helping surgeons to gain a deeper understanding in the complex pathomorphology of the diseased heart. Future studies are suggested to quantitatively measure whether the use of 3DPHM reduces the operative time, hospital admission duration, as well as morbidity and mortality rate. From there, a cost-benefit analysis can be carried out to evaluate if 3D printing of CHD is worthwhile in the healthcare industry.

The clinical value of 3DPHM in enhancing communication in clinical practice is arguable. Even though both health professionals and non-professionals are satisfied with the use of 3DPHM during the consultation, there is a lack of quantitative evidence to suggest the increase in parental knowledge with the use of 3DPHM, nor is there evidence to suggest the reduction in consultation time. However, with only 5 studies that investigated the utility of 3DPHM in this area, a solid conclusion could not be drawn. Future studies should aim to measure the impact of 3DPHM on the reduction in consultation time, as well as the knowledge acquisition among the patients and parents.

Last but not least, a comprehensive cost-benefit analysis for implementation of 3D printing technology in cardiovascular surgeries lacks in the current literature and this needs to be addressed by future studies. 

## Figures and Tables

**Table 1:** Characteristics of the included studies.

First Author/Year	Study Design	CHD Types	Imaging Modality	Segmentation Software	Utility
* Lau et al. 2018 [2]	Cross-sectional	DORV with sub-aortic VSD	CTA	Mimics	Accuracy, pre-operative planning, communication, medical education
* Ma et al. 2015 [5]	Case series	ToF, ToF with ASD, ToF with PDA	CTA	Philips EBW Comp-Cardiac post-processing software	Accuracy, intraoperative orientation, impact on patients’ outcomes ^a^
* Riesenkampff et al. 2009 [6]	Case series	DORV, VSD, LVOTO, CoA, RVOTO, AVSD, pulmonary atresia, pulmonary stenosis, TGA, congenitally corrected TGA	CTA/CMR	Medical Imaging and Interaction Toolkit	Pre-operative planning
* Schmauss et al. 2015 [7]	Case series	subpulmonary VSD, HLHS, pulmonary atresia and hypoplastic right ventricle, aortic stenosis	CTA/CMR	Amira, MeVisLab-Environment	Pre-operative planning, intraoperative orientation, pre-surgical simulation
* Shiraishi et al. 2009 [8]	Case series	CoA, DORV, VSD, HLHS	CTA	NR	Pre-operative planning, pre-surgical simulation
* Valverde et al. 2017 [9]	Prospective case-crossover	DORV, Complex TGA, univentricle, VSD, criss-cross heart, LVOTO, discordant AV and VA connections	CTA/CMR	ITK Snap	Accuracy, pre-operative planning, communication, medical education
* Bhatla et al. 2017 [10]	Case series	Complex muscular VSD, DORV	CTA/CMR	Mimics	Pre-operative planning
* Ejaz et al. 2013 [16]	RCT	NR	CTA	Advance Workstation (GE Health Systems), Mimics	Medical education, pre-operative planning
* Garekar et al. 2016 [17]	Case series	DORV with remote VSD	CTA/CMR	NR	Pre-operative planning
* Hoashi et al. 2018 [18]	Case series	DORV, TGA, congenitally corrected TGA, interrupted aortic arch Type B, ToF and MAPCA, HLHS, functional single ventricle, mitral stenosis, AVSD	CTA	NR	Pre-operative planning, pre-surgical simulation
* Loke et al. 2017 [19]	RCT	Unrepaired ToF, repaired ToF	CTA/CMR/echocardiography	Mimics	Medical education
* McGovern et al. 2017 [20]	Case series	univentricular heart, abnormal systemic or pulmonary venous drainage, dextrocardia, TGA, HLHS	CTA	Mimics	Pre-operative planning
* Ngan et al. 2006 [21]	Case series	VSD, pulmonary atresia, MAPCA	CTA	Mimics	Pre-operative planning
* Olejnik et al. 2017 [22]	Case series	interrupted aortic arch type A with aortopulmonary window type 2, dextroversion, DORV with subaortic VSD, CoA, ToF	CTA	3D Slicer	Accuracy, pre-operative planning
* Olivieri et al. 2015 [23]	Case series	VSD	echocardiography	Mimics	Accuracy
* Olivieri et al. 2016 [24]	Cross-sectional	HLHS with total anomalous pulmonary venous connection, supravalvar aortic stenosis, DORV with hypoplastic and stenotic aortic valve and hypoplastic aortic arch, aortic regurgitation, right partial anomalous pulmonary venous connection, left pulmonary artery sling, RVOTO, truncal valve regurgitation, double aortic arch, TGA with VSD and pulmonary atresia	CTA/CMR	Mimics	Medical education
* Parimi et al. 2018 [25]	Case series	HLHS post Glenn shunt, CoA, ToF with MAPCAs, pulmonary atresia	Rotational angiography	Osirix	Accuracy
* Ryan et al. 2018 [26]	Case control study	pulmonary atresia, ToF, DORV, truncus arteriosus, single ventricle	CTA/CMR	Mimics	Pre-operative planning, impact on patients’ outcomes
* Su et al. 2018 [27]	RCT	3 different subtypes of VSD	CTA	NR	Medical education
* Wang et al. 2017 [28]	RCT	VSD, pulmonary atresia, MAPCA	CTA	Mimics	Medical education
* White et al. 2018 [29]	RCT	3 different subtypes of VSD, ToF	NR	Philips IntelliSpace Portal	Medical education
* Zhao et al. 2018 [30]	Cross-sectional	DORV	CTA	Mimics	Accuracy, pre-operative planning, impact on patients’ outcomes
* Biglino et al. 2017a [4]	Pre-post study	ToF, TGA, CoA, pulmonary atresia, aortic stenosis with dilated ascending aorta, DORV, Ebstein’s anomaly	CMR	Simpleware	Communication
Biglino et al. 2015a [11]	RCT	CoA, pulmonary atresia, ToF, TGA, aortic stenosis, bicuspid aortic valve, total anomalous pulmonary venous drainage, double-inlet left ventricle	CMR	Mimics	Communication
Biglino et al. 2015b [12]	Cross-sectional	TGA, ToF, pulmonary atresia, CoA, HLHS, TCPC	CMR	Mimics	Pre-operative planning, medical education, communication
Biglino et al. 2017b [13]	Cross-sectional	repaired TGA, CoA, ToF, pulmonary atresia with intact ventricular septum, palliated HLHS	CMR	NR	Medical education
* Costello et al.2015 [15]	Pre-post study	5 different subtypes of VSD	CMR	Mimics	Medical education
Costello et al. 2014 [14]	Pre-post study	5 different subtypes of VSD	CMR	Mimics	Medical education

* = articles that were included in the review. ^a^ patients’ outcome includes length of cardiopulmonary bypass time, aortic cross-clamp time, mechanical ventilation time, duration of the surgery, patients’ readmission rate, length of hospitalization, and mortality rate. 3DPHM, three-dimensional printed heart models; AV, atrio-ventricular; AVSD, atrio-ventricular septal defect; ASD, atrial septal defect; CHD, congenital heart disease; CMR, cardiac magnetic resonance; CoA, coarctation of aorta; CTA, computed tomography angiography; DORV, double outlet right ventricle; HLHS, hypoplastic left heart syndrome; LVOTO, left ventricular outflow tract obstruction; MAPCA, major aortopulmonary collateral arteries; NR, not reported; PDA, patent ductus arteriosus; RCT, randomized controlled trial; RVOTO, right ventricular outflow tract obstruction; SVC, superior vena cava; TCPC, total cavopulmonary connection; TGA, transposition of great arteries; ToF, Tetralogy of Fallot; VA, ventriculoarterial; VSD, ventricular septal defect.

**Table 2:** Quality of the included studies assessed by National Institute of Health assessment tools.

Studies	Quality Rating
Lau et al. 2018 [2]	Fair
Biglino et al. 2017a [4]	Fair
Ma et al. 2015 [5]	Good
Riesenkampff et al. 2009 [6]	Fair
Schmauss et al. 2015 [7]	Good
Shiraishi et al. 2009 [8]	Fair
Valverde et al. 2017 [9]	Good
Bhatla et al. 2017 [10]	Good
Costello et al. 2015 [15]	Fair
Ejaz et al. 2013 [16]	Fair
Garekar et al. 2016 [17]	Good
Hoashi et al. 2018 [18]	Good
Loke et al. 2017 [19]	Fair
McGovern et al. 2017 [20]	Good
Ngan et al. 2006 [21]	Good
Olejnik et al. 2017 [22]	Good
Olivieri et al. 2015 [23]	Good
Olivieri et al. 2016 [24]	Fair
Parimi et al. 2018 [25]	Good
Ryan et al. 2018 [26]	Good
Su et al. 2018 [27]	Good
Wang et al. 2017 [28]	Fair
White et al. 2018 [29]	Good
Zhao et al. 2018 [30]	Fair

## Supplementary Materials

The supplementary materials are available online at https://www.mdpi.com/2077-0383/8/9/1483/s1.
