# Supplementary material for: Dimensional Accuracy and Clinical Value of 3D Printed Models in Congenital Heart Disease: A Systematic Review and Meta-Analysis
Source: J Clin Med. 2019 Sep 18;8(9):1483. doi: 10.3390/jcm8091483 (PMC6780783; doi:10.3390/jcm8091483)
Supplement: Supplementary file 1 [file jcm-08-01483-s001.pdf]

Supplementary Table S1: Quality assessment of RCTs

Items	Ejaz 2013	Loke 2017	Su 2018	Wang 2017	White 2018
Described as RCT	Yes	Yes	Yes	Yes	Yes
Adequate randomization	No	No	Yes	No	Yes
Treatment allocation concealed	No	No	Yes	No	Yes
Participants and providers blinded	Yes	No	Yes	Yes	Yes
Outcomes assessors blinded	No	No	No	No	Yes
Similarity of groups at baseline	No	Yes	Yes	No	Yes
Drop-out rate < 20%	Yes	Yes	Yes	Yes	Yes
Differential drop-out rate < 15%	Yes	Yes	Yes	Yes	Yes
Adherence to intervention protocols	Yes	Yes	Yes	Yes	Yes
Avoid other interventions	N/A	N/A	N/A	N/A	Yes
Outcomes measures assessment	Yes	Yes	Yes	No	Yes
Power calculation	No	No	No	No	No
Prespecified outcomes	Yes	Yes	Yes	Yes	Yes
Intent-to-treat analysis	Yes	Yes	Yes	Yes	Yes
Total of 'Yes'	8	8	11	7	13
Quality rating	Fair	Fair	Good	Fair	Good

RCT, randomised controlled trial; N/A, not available

Supplementary Table S2: Quality assessment of observational cohort and cross-sectional studies

Items	Biglino 2017a	Lau 2018	Olivieri 2016	Valverde 2017	Zhao 2018
Research question	Yes	Yes	Yes	Yes	Yes
Study population specified	Yes	Yes	Yes	Yes	Yes
Participation rate >50%	Yes	Yes	Yes	Yes	Yes
Groups recruited from the same population/ uniform eligibility criteria	Yes	Yes	Yes	Yes	Yes
Sample size justification	No	Yes	No	No	No
Exposure assessed before outcome measurement	Yes	Yes	Yes	Yes	Yes
Sufficient timeframe to see an effect	No	No	No	Yes	No
Different levels of exposure examined	No	No	No	Yes	No
Exposure measures and assessment	Yes	Yes	Yes	Yes	Yes
Repeated exposure assessment	No	No	Yes	No	No
Outcomes measures	Yes	Yes	Yes	Yes	Yes
Outcomes assessors blinded	No	No	No	No	No
Follow-up rate	Yes	Yes	Yes	Yes	Yes
Statistical analyses	No	No	No	No	No
Total of 'Yes'	8	9	9	10	8
Quality rating	Fair	Fair	Fair	Good	Fair

Supplementary Table S3: Quality assessment of pre-post studies with no control group

Items	Costello 2015
Research question	Yes
Eligibility criteria and study population	No
Study participants representative of clinical populations of interest	Yes
All eligible participants enrolled	No
Sample size	No
Intervention clearly described	Yes
Outcome measures clearly described, valid, and reliable	Yes
Outcomes assessors blinded	No
Follow-up rate	Yes
Statistical analyses	Yes
Multiple outcomes measures	No
Group-level interventions and individual-level outcome efforts	Yes
Total of 'Yes'	7
Quality rating	Fair

Supplementary Table S4: Quality assessment of case-control study

Items	Ryan 2018
Research question	Yes
Study population	Yes
Target population and case representation	No
Sample size justification	Yes
Groups recruited from the same population	Yes
Inclusion/exclusion criteria	Yes
Case and control definitions	No
Random selection of study participants	No
Concurrent controls	Yes
Exposure assessed before outcome measurement	Yes
Exposure assessors blinded	No
Statistical analyses	No
Total of ‘Yes’	9
Quality rating	Good

Suppplementary Table S5: Quality assessment of case series studies

Items	Bhatla 2017	Garekar 2016	Hoashi 2018	Ma 2015	McGovern 2017	Ngan 2006	Olejnik 2017	Olivieri 2015	Parimi 2018	Riesenkampff 2009	Schmauss 2015	Shiraishi 2009
Research question	Yes	Yes	Yes	Yes	Yes	Yes	Yes	Yes	Yes	Yes	Yes	Yes
Study population clearly described	Yes	Yes	Yes	Yes	Yes	Yes	Yes	Yes	Yes	Yes	Yes	Yes
Case consecutive	Yes	Yes	Yes	Yes	Yes	Yes	Yes	Yes	Yes	No	Yes	No
Comparable subjects	Yes	Yes	Yes	Yes	Yes	Yes	Yes	Yes	Yes	Yes	Yes	Yes
Intervention clearly described	Yes	Yes	Yes	Yes	Yes	Yes	Yes	Yes	Yes	Yes	Yes	Yes
Outcome measures clearly described, valid, and reliable	Yes	Yes	Yes	Yes	Yes	Yes	Yes	Yes	Yes	Yes	Yes	No
Follow-up rate	Yes	Yes	Yes	Yes	Yes	Yes	Yes	No	No	No	Yes	No
Statistical methods described	No	No	No	Yes	No	No	Yes	Yes	Yes	No	No	No
Results well described	Yes	Yes	Yes	Yes	Yes	Yes	Yes	Yes	Yes	Yes	Yes	No
Total of ‘Yes’	8	8	8	9	8	8	9	8	8	6	8	4
Quality rating	Good	Good	Good	Good	Good	Good	Good	Good	Good	Fair	Good	Fair
